# RACE AND PLACE: PHYSICAL PERFORMANCE DECLINES IN BLACK AND WHITE OLDER ADULTS IN FOUR US COMMUNITIES

**DOI:** 10.1093/geroni/igad104.1967

**Published:** 2023-12-21

**Authors:** B Gwen Windham, William Windham, Hunter Sylvester, A Richey Sharrett, Laura Skow, Priya Palta, Anna Kucharska-Newton, Michael Griswold

**Affiliations:** UMMC-The MIND Center, Jackson, Mississippi, United States; University of Mississippi School of Medicine, Jackson, Mississippi, United States; UMMC-The MIND Center, Jackson, Mississippi, United States; Johns Hopkins Bloomberg School of Public Health, Baltimore, Maryland, United States; Johns Hopkins Bloomberg School of Public Health, Baltimore, Maryland, United States; UNC Gillings School of Global Public Health, Chapel Hill, North Carolina, United States; UNC Gillings School of Global Public Health, Chapel Hill, North Carolina, United States; UMMC-The MIND Center, Jackson, Mississippi, United States

## Abstract

Studies suggest disparities in physical function between Black and White adults, but most lack longitudinal, objective functional measures across race and geographic regions. We examined Short Physical Performance Battery (SPPB) changes among 5,836 Black and White older adults (>65 years, 22% Black, 41% men) with up to three SPPB assessments. Exams were conducted in the Atherosclerosis Risk in Communities Study (Visit 5 (2011-13)-Visit 7 (2017-19), median follow-up 5.8 years (max 8.4)) in four US communities: North Carolina (n=1248; 7% Black), Mississippi (n=1179, 100% Black), Maryland (n=1618; 0.9% Black) and Minnesota (N=1791; n=0.5% Black). A difference of 0.5 SPPB points (scored 0-12) is clinically meaningful. SPPB declines were estimated using marginal standardization following GEE (with log-links, negative binomial distributions, exchangeable covariance, robust SE, adjusting for demographics, BMI, diabetes, hypertension, smoking, drinking status, heart failure, heart disease, and stroke). SPPB declines were 3.4 times greater among Black versus White participants overall; -1.99 versus -0.59 points; absolute difference=-1.41 (95% CI: -1.67,-1.14), relative difference=3.40 (2.65, 4.15). However, some within-race, between-site differences were similar; Maryland-White SPPB declines were 3.8 (2.3, 6.3) times greater than North Carolina-White SPPB declines, and Mississippi-Black declines were 2.5 (1.10, 5.90) times greater than North Carolina-Black declines. Furthermore, between-race (Black versus White) differences were less supported statistically within the same site; SPPB declines were 2.2 (0.85, 5.80) greater in Black than White participants in North Carolina. This suggests that regional variations in physical function declines may be at least as strong as differences attributed to race; both are important to consider.

